# Dr. John T. Metcalfe

**Published:** 1902-03

**Authors:** 


Dr. John T. Metcalfe, formerly of New York, died at his home in Thomasville, Ga., January 30, 1902, aged 84 years. He was for many years one of New York's most noted physicians and a teacher of medicine at the College of Physicians and Surgeons.

